# Assessing the Usability of ChatGPT for Formal English Language Learning

**DOI:** 10.3390/ejihpe13090140

**Published:** 2023-09-21

**Authors:** Sarang Shaikh, Sule Yildirim Yayilgan, Blanka Klimova, Marcel Pikhart

**Affiliations:** 1 Department of Information Security and Communication Technology (IIK), Norwegian University of Science and Technology (NTNU), 2815 Gjøvik, Norway; sarang.shaikh@ntnu.no (S.S.); sule.yildirim@ntnu.no (S.Y.Y.); 2 Department of Applied Linguistics, Faculty of Informatics and Management, University of Hradec Kralove, Rokitanskeho 62, 500 03 Hradec Kralove, Czech Republic; blanka.klimova@uhk.cz

**Keywords:** formal learning, English language, chatbots, ChatGPT, AI, usability testing, language learning

## Abstract

Recently, the emerging technologies have been constantly shaping the education domain, especially the use of artificial intelligence (AI) for language learning, which has attracted significant attention. Many of the AI tools are being used for learning foreign languages, in both formal and informal ways. There are many studies that have explored the potential of the recent technology “ChatGPT” for education and learning languages, but none of the existing studies have conducted any exploratory study for assessing the usability of ChatGPT. This paper conducts an assessment for usability of ChatGPT for formal English language learning. The study uses a standard questionnaire-based approach to ask participants about their feedback for usefulness and effectiveness of ChatGPT. The participants were asked for their feedback after performing series of tasks related to formal English language learning with ChatGPT. A variety of student participants were selected for this study with diverse English language proficiency levels, education levels, and nationalities. The quantitative analysis of the participant responses shed light on their experience with regards to the usability of ChatGPT for performing different English language learning tasks such as conversation, writing, grammar, and vocabulary. The findings from this study are quite promising and indicate that ChatGPT is an effective tool to be used for formal English language learning. Overall, this study contributes to the fast-growing research domain on using emerging technologies for formal English language learning by conducting in-depth assessment of usability for ChatGPT in formal English language learning.

## 1. Introduction

The English language is recognized as the lingua franca [1] due to its use in various fields, such as education [2], business [3], and entertainment [4]. Therefore, being more proficient in the English language can help in obtaining more job opportunities, facilitating professional and academic communication, and improving the English language knowledge. The formal learning of the English language provides a structured way to help learners to develop their language skills, such as knowing and using grammar rules, vocabulary, and language functions (reading, writing, conversating, listening, etc.) more efficiently. The most common structured ways to learn formal English language are taking part in group discussions, debates, and giving presentations, which improve the learners’ speaking, reading, writing, and listening skills.

To learn a language formally is an important skill in today’s global world, and emerging technologies have created new opportunities for language learners [5]. With the popularity of one of the emerging technologies, namely “Chatbots”, there has been strong increasing interest to use their potential for formal language learning, especially English [6]. The chatbot technology provides a conversational environment where a user can interact in a way like interacting with a human [7]. The chatbot can be queried by a user for certain information, and it can provide feedback to the user on various language aspects, such as grammar or vocabulary, when explicitly asked to do so. In this case, it can be used for English language learning [8]. For example, Jeon in his empirical study [7] showed that chatbots for language learning could not only promote vocabulary acquisition but could also offer diagnostic information about individual learners concerning vocabulary learning. In addition, as research in Computer-Assisted Language Learning (CALL) indicates, there is a consensus that especially rule-based, scripted voice systems are optimal for language learning [9].

However, effectiveness of the chatbots for language learning highly depends on their usability, efficiency, and effectiveness [10]. The term usability can be defined as “the metric to which a program can be used to achieve quantified objectives with effectiveness, efficiency, and satisfaction in a specified context of use” [11]. 

Recently, ChatGPT has evolved as the most advanced conversational AI-based tool developed by OpenAI on the top of GPT-3.5 architecture. The term GPT stands for “Generative Pre-trained Transformer”. It is a state-of-the-art large language model that is trained using a large amount of text data, which helps it to generate human-like contextual text responses. ChatGPT can easily understand the text input and generate the contextual response accordingly. ChatGPT produces the responses based on the learned patterns from the training data and not from any specific source or real-time information. Although the aim is to give users contextually correct, relevant responses, it may also generate incorrect answers. ChatGPT is fine-tuned on the basis of a dialogue dataset such that the generated responses could be in form of conversation with back-and-forth interactions between the user and model [12]. 

The purpose of current research study is the assessment of the usability of ChatGPT for learning language, specifically for English language, in a formal way. In this paper, usability is defined as “the metric to which a program can be used to achieve quantified objectives with effectiveness, efficiency, and satisfaction in a specified context of use” [11]. The following are the research problem and the objective of the current study.

Research Question: What is the potential usability of ChatGPT as a tool for formal English language learning?

Objective: To evaluate the potential of ChatGPT for English language learning in formal settings, by measuring its usability through exploring learners’ satisfaction levels with the responses generated by ChatGPT and learners’ perceptions and experiences about its ease of use, usefulness, potential benefits, and limitations while learners perform English language learning tasks namely writing, conversation, grammar, and vocabulary.

## 2. Literature Review

Language learning is the process of acquiring relevant knowledge and skills for any foreign or second language, in this case English language, which enables individuals to communicate and interact with speakers of that language in an effective manner. Generally, it consists of developing proficient skills in various language components such as writing, reading, speaking, and listening [13]. Learning a foreign language is important for several reasons such as communication, personal and professional opportunities, and cognitive development.

Traditional digital language learning technologies (TDLLT) are the computer/software programs designed for language learning using various digital platforms such as desktop software programs, online websites, mobile applications, etc. These technologies have been widely used for the past several years and are recognized for their effectiveness in language learning [14]. Some of the common TDLLT examples are listed in Table 1 along with their description and purpose.

Recently, emerging language learning technologies (ELLT) are the most recent and innovative tools, which utilize the power of new techniques to improve the language learning experience. These technologies are built on top of the advanced techniques like virtual reality (VR), artificial intelligence (AI), and natural language processing (NLP), gamification, etc. These technologies are more advanced to give a more immersive, effective, and personalized language learning experience [5]. Some of the common ELLT examples are listed in Table 2 along with their description and purpose.

This current study is focused on the assessment of ChatGPT for formal English language learning and ChatGPT tool comes under the umbrella of chatbots and conversational AI. Therefore, this study will only focus on the existing studies combining chatbots and learning languages.

Recently, chatbots have gained a lot of familiarity for language learning due to their ability to perform one-on-one conversation with the users using natural language processing (NLP) in any target language [28,29]. Different users use chatbots for daily language learning and practice, such as conversation, asking/generating questions, and conducting assessments like vocabulary tests [30]. Research also shows that such technology can offer different types of interactional exercises that can encourage learners to produce more output in a low-anxiety environment [29,31]. The most used chatbots in language learning context consists of mainly three common features: (1) the availability to support users/students 24/7 since a human partner cannot do it easily all the time, (2) the availability of broad language information, which human partners might lack, and (3) to play the role of an assistant to perform repetitive work such as answering frequent questions and to practice the language. 

The authors in [28] performed a systematic literature study for using chatbots for language learning where the authors identified the English language as the most dominant language in the use of chatbots specifically for language learning. Furthermore, the authors also reported that the most common tasks performed by users for learning language with chatbots were speaking, listening, writing, reading, practicing vocabulary and grammar, and making conversation. Most of the studies reported the use of chatbots for language learning by students in higher education (e.g., undergraduate and postgraduate students). However, there are also studies focusing on primary and secondary school students, such as [29,32], which shows a positive impact on students acquiring English as a foreign language.

Okonkwo and Ade-Ibijola [33] conducted a systematic review study on the use of chatbots in education where they reported the maximum number of the studies being part of the “Teaching and Learning” domain (66%) along with other domains such as “Research and Development” (19%), “Advisory” (4%), “Assessment” (6%), and “Administration” (5%). The major studies linked with the use of chatbots for teaching and learning are discussed in [34,35,36]. 

However, despite of the many advancements for using emerging technologies in language learning, the effectiveness, user experiences, satisfaction, and user engagement with these technologies are still an open problem. The authors in [37] conducted a meta-analysis for using mobile applications in learning English language. At first, the authors confirmed based on the meta-analysis that using mobile applications for learning English language are valid alternate options as compared to traditional technologies such as flash cards, etc. In addition to this, the authors concluded the same for all education levels used in the meta-analysis with the maximum learning for being at the bachelor’s level. Finally, the authors concluded the study with the statement that mobile apps-based language learning gives better results than traditional lecture-based setting.

**Figure 1:**
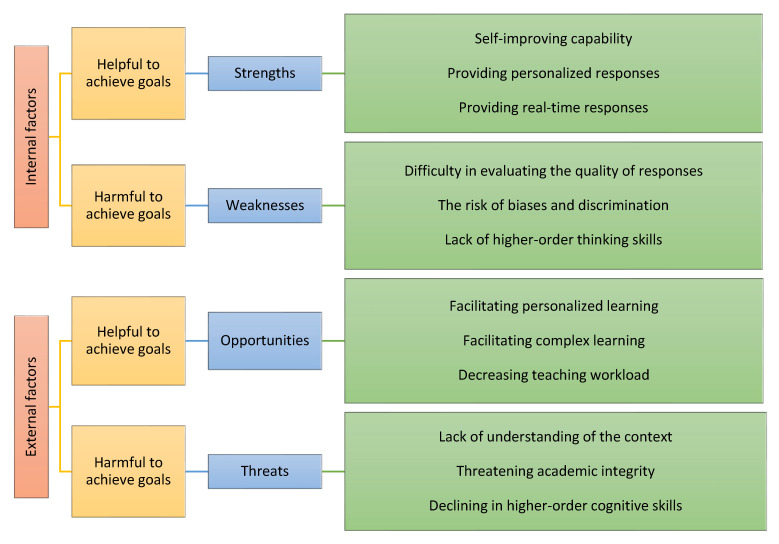
SWOT analysis of ChatGPT in language learning and education [39].

The authors in [38] performed a systematic review of using gamification tools for foreign language learning. The authors put focus on understanding the effectiveness of the gamification tools as according to them the features of gamification tools for learning language are not well understood in existing studies. The authors identified a list of different positive and negative impacts as well as no impacts of gamification techniques for foreign language learning. Additionally, the authors identified the list of factors that affect the effectiveness of these tools. Furthermore, the authors in [39] performed a “SWOT (Strengths, Weaknesses, Opportunities, and Threats)” analysis of ChatGPT for language learning and education. Figure 1 shows some of the major points in each category of the SWOT evaluation conducted in this study. As we can see from the figure, along with many strengths and opportunities associated with the use of ChatGPT, there are still many weaknesses and threats identified on ChatGPT that could lead to the misuse of it for language learning.

Although existing state-of-the-art studies have discussed the role of ChatGPT for language learning and its implications, none of them have performed any exploratory study/work with the actual use of ChatGPT for language learning. Furthermore, there exists no such study that assesses the usability of ChatGPT for language learning either formal or informal. Therefore, the scope of this research study is to explore and assess the usability of ChatGPT for formal English language learning. To start with assessing the usability of ChatGPT for formal English language learning, we planned a physical research activity at NTNU premises where a set of participants can perform several tasks with ChatGPT and give their feedback regarding their experience.

## 3. Methodology

**Figure 2:**
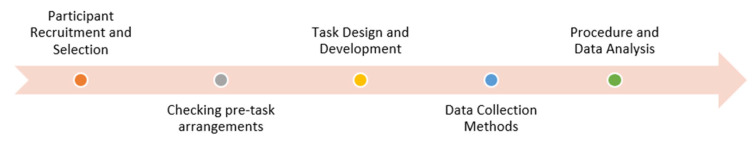
An outline of the methodology procedure.

Figure 2 shows the list of steps involved in the overall methodology. Each of the steps is explained in detail in the next subsequent sections.

### 3.1. Participants

A total of 10 participants were recruited for this study, which corresponds to the well-established methodology, namely usability testing described in the Introductory part, and which follows the rule of only 5 users. The recruitment process consisted of disseminating regarding the research activity through the official communication channels of the “Norwegian University of Science and Technology (NTNU), Gjøvik”. The target participants for this activity were exclusively students enrolled in any department of the university. The dissemination post regarding the research activity is available at the link (https://shorturl.at/kuwS8) (accessed on 1 August 2023). Participant information, such as email, country, and current study program, was collected from each participant using an online registration form. The data were collected in a confidential manner to protect participant privacy and anonymity.

Before registering for the activity, in the online dissemination announcement, all the participants were provided with detailed information about the research group organizing the study, the relevant project, the aim of the study, and the details of designed tasks. To ensure the privacy of the participants and the confidentiality of the data to be collected, each participant was assigned a random ID number automatically in the online registration form. The data were stored in a secure manner and were only accessible to the team involved in this study. The ethical guidelines and regulations were followed in compliance with The Research Ethics Committee of NTNU (https://shorturl.at/joFH0) (accessed on 1 August 2023). The research process conducted for this study was carefully reviewed and approved by the Committee for Research Ethics of the University of Hradec Kralove, Czech Republic, no. 4/2023. All required GDPR standards regarding privacy and data protection were strictly followed. 

### 3.2. Data Collection

The usability testing activity took place in a controlled meeting room environment with sufficient capacity for 10–15 persons. The participants interacted with the ChatGPT tool using their own laptop device and email account. Stable internet connection was ensured to make participants have smooth communication and experience.

The task design and development steps were carefully carried out to evaluate ChatGPT’s usability for formal English language learning. There were, in total, four modules in the overall designed task mentioned above: (1) The participants were instructed to have conversations with ChatGPT on different topics. (2) The participants asked ChatGPT to write paragraphs for different contexts such as the context of formal writing, the context of informal writing, etc. (3) The participants were instructed to try ChatGPT for identifying, fixing and getting suggestions for fixing grammar mistakes, and (4) The participants were instructed to practice vocabulary learning with ChatGPT. The total time given to each participant to perform all these 4 modules was 1 h (i.e., 15 min for each module). These tasks were designed to cover a range of language learning skills and to understand the usability of ChatGPT. Appendix A.1 shows the details of the tasks we carried out with all the participants of the activity.

We developed a post-task questionnaire to ask participants for their feedback/experience regarding the usability of ChatGPT and their overall experience during the 1 h of interaction with the tool. The questionnaire was comprised of 5 major parts: (1) Demographics information, (2) Previous English language knowledge level, (3) Feedback using “Usefulness, Satisfaction, and Ease of Use (USE)” Questionnaire, (4) Feedback using “System Usability Scale (SUS)” Questionnaire, (5) Satisfaction with tasks performed (i.e., conversation, writing, grammar, and vocabulary). The complete questionnaire is available in the Appendix A.2. The next two subsections will show some details regarding the USE and SUS questionnaires.

The USE questionnaire (https://garyperlman.com/quest/quest.cgi?form=USE) (accessed on 1 August 2023) is a common tool that is used for the evaluation of usability of a technical system. It consists of a series of 30 statements or items for which users respond on a Likert scale from 1 to 5 by showing their disagreement or agreement. A score of 1 is strong disagreement and 5 is strong agreement on the scale. This questionnaires’ goal is to assess users’ perceptions and experience for the system’s overall usability for performing any specific task [40].

The SUS questionnaire (https://measuringu.com/sus/) (accessed on 1 August 2023) is again a commonly used tool for measuring the “perceived usability” of a technical system. Again, it consists of a series of ten statements or items for which participants respond on a Likert scale from 1 to 5 by showing their agreement or disagreement. A score of 1 is strong disagreement and 5 is strong agreement on the scale. This questionnaire gives a standardized and reliable measure for assessing usability and satisfaction of a system for performing any task [40].

### 3.3. Data Analysis

For the data analysis part, to obtain maximum insights regarding the usability of ChatGPT for our work, we decided to analyze the participants’ responses for SUS and USE questionnaire questions and link those with the performance of ChatGPT to generate responses to user tasks as well as with the participants’ English language skill level and demographics. To be more concise, while performing the data analysis, we selected some of the standard statistical measures/methods to extract insights from the participants’ responses from the post-experiment questionnaire form. The chosen methods are mean, standard deviation, skewness, and kurtosis.

## 4. Results

The responses from the 10 participants were analyzed. The 10 participants provide a sufficient sample for our study purpose shows the distribution of participants’ demographics based on different attributes such as age, gender, education level, and department. For this study, we specifically focused on the participants who are students and currently enrolled in any program at NTNU.

**Figure 3:**
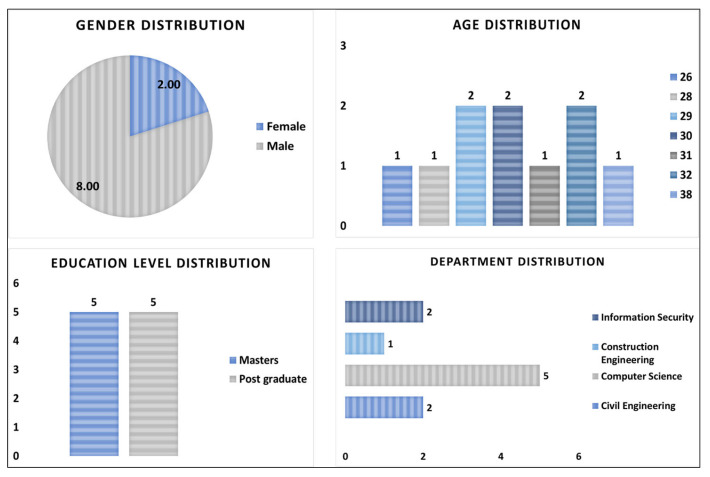
Participants’ demographics statistics.

In Figure 3, the sample population consisted of 80% male participants and 20% female participants. The age distribution for the participants is ranging from 26 to 38 years, with a mean age value of 30 years. Most of the participants lie within the 26–30 age group (60%), followed by the 31–34 age group (30%) and the 35–38 age group (10%). In the current education level distribution, the number of participants remained equal between masters (50%) and postgraduate (50%) education levels. The participants also represented a wide range of distribution among different departments to which they belong. Most of the participants/students belong to the Computer Science department (50%), followed by the Civil Engineering, Information Security departments (20% each), and the Construction Engineering department (10%). The study involved participants from various nationalities of the world. Out of total 10, 25% of the participants were Norwegians, followed by participants from Italy, Georgia, Nepal, and Pakistan.

The English language skill level of the participants was acquired in the post-experiment questionnaire form where each of the participants rated himself/herself on a scale from 1 to 5 (1 being minimum and 5 being maximum skill level). The participants performed ratings for four different language skills (i.e., listening, writing, speaking, and reading). The results from these ratings provide insights into participants’ overall English language skill levels. The descriptive statistics of participants’ English language skill levels are shown in Table 3 in the form of mean, SD, min, max, skewness, and kurtosis values for each of the English language skills.

The mean score of the participants’ English language skill level for each skill was found to be 3.50 (Speaking), 3.70 (Listening), 4.30 (Reading), and 3.80 (Writing) out of 5. This shows an indication of moderately high level of English language competence of the participants. Furthermore, the minimum and maximum language skill level for all four skills was found to be 2 and 5, respectively. We also calculated the standard deviation (SD) for all the language skills levels of the whole group of participants. Overall, the SD was found to be 0.84, 0.94, 0.94, and 0.91 for speaking, listening, reading, and writing language skills, respectively. These values indicate that there is a moderate variability in the participants’ responses where the data points are spread out from the mean but not in an excessive manner. 

Additionally, we also analyzed the skewness and kurtosis statistical measures to understand the patterns of participants’ responses for their English language skill level. The skewness distribution of the speaking level is completely symmetric as the value is 0, which shows a normal distribution of participants for the rating scale from 1 to 5. However, the skewness distribution of rest of the three English language skills, listening (−0.23), reading (−1.71), and writing (−0.60) does not show a normal distribution among the participants. However, the values are left-skewed, which shows that the majority of the participants are closer to the higher end of the scale (closer to 5) as compared to the lower end of the scale (close to 1) pointing to higher language skill levels for these three skills. The kurtosis distribution for the speaking and writing levels show the normal distribution among the participant responses as the values are 0.10 and 0.39, respectively. This shows that the peak of the distribution is neither too sharp nor too flat as compared to the normal distribution. Furthermore, the remaining two kurtosis values for listening (−0.34) and reading (3.53) do not show a normal distribution among the participants. The value −0.34 shows the relatively flat peak/flatter distribution than normal distribution indicating a lighter-tailed distribution. Lastly, the value 3.53 shows a higher peaked concentration of data points around the overall mean as compared to the normal distribution.

**Figure 4:**
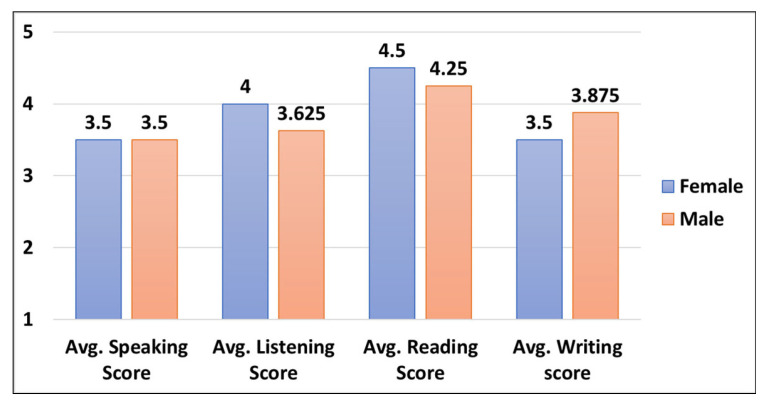
Gender-based participants English language skills level scores.

Figure 4 shows the distribution of each English language skill level average scores based on the gender attribute. The female group is dominating the average scores for three English language skills, speaking, listening, and reading with an average score of 3.5, 4, and 4.5, respectively as compared to the male group. The remaining writing skill level is dominated by the male group as compared to the female group with an average score of 3.87.

**Figure 5:**
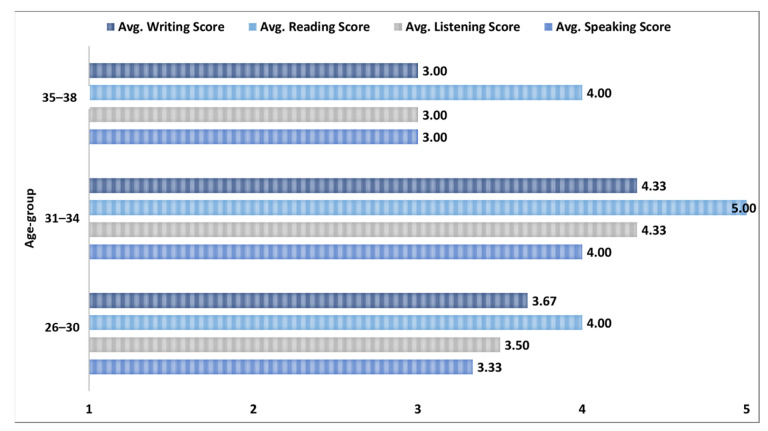
Age group versus English language skills level scores.

Figure 5 depicts the English language skills level distribution based on three age groups. We originally had only the age of each participant, and based on the age values we created three age groups (i.e., 26–30, 31–34, and 35–38). The age group 31–34 has the highest average scores of all the English language skill levels followed by the 26–30, 35–38 age groups, respectively. For the age group 31–34, the highest score for participants is in reading (5) followed by writing (4.33), listening (4.33), and speaking (4). The age-group 26–30, which consists of the 60% of the participants, also has the maximum average score for reading (4) followed by the writing (3.67), listening (3.50), and speaking (3.33). The age group that has the lowest average scores is the 35–38 group where the reading level has the highest average score (4) and rest of the English language skills levels have equal scores (3). The average score distribution for all four tasks for this group is also slightly different in comparison to the other two age groups. 

**Figure 6:**
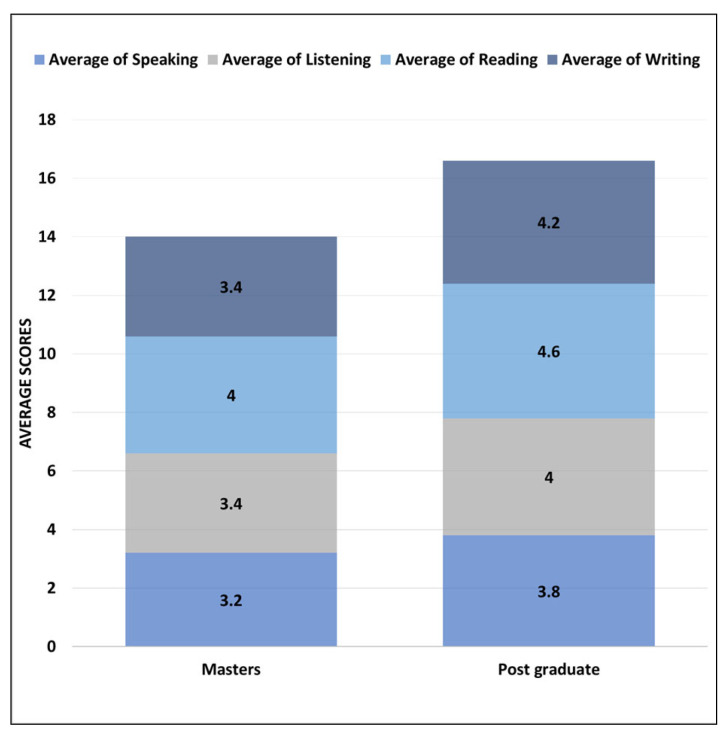
Education-level versus English language skills levels scores.

Figure 6 shows the distribution of average scores for different English language skill levels divided into the participants’ current education level. Overall, the postgraduate group has the highest scores in all the language skills as compared to the masters group. The reading skills of the participant is again leading here for both groups (i.e., masters and postgraduate) as compared to the remaining three English language skill levels. The lowest average scores in all the language skills seems to be for the speaking skill for both groups.

Next, we explain the quantitative scores of participants’ rating for each of the task categories, which the participants performed with ChatGPT. We already discussed these tasks in Section 3.3 (i.e., conversation, writing, grammar, and vocabulary). The ratings for these tasks were acquired in the post-experiment questionnaire form where each of the participants rated their experience with each of the tasks performed (i.e., conversation, writing, grammar, and vocabulary) with ChatGPT. The ratings were in the range from 1 to 5 (i.e., 1 being minimum and 5 being maximum skill level). The results from these ratings provide insights into participants’ experience with ChatGPT while performing the given tasks. The descriptive statistics of participants’ task performance ratings are shown in Table 4. where we have shown the mean, SD, min, max, skewness, and kurtosis values for each of the task categories.

The mean score for ChatGPT performance on each of the task categories was found to be 3.90 (Conversation), 4.10 (Writing), 4.20 (Grammar), and 4.30 (Vocabulary) out of 5 as identified from the participants ratings. This is an indication of a very good performance of ChatGPT in response to all of the four tasks performed by the participants mentioned above in the document. Furthermore, the minimum and maximum rating for the performance for all the tasks was found to be 2 and 5, respectively, with an exception where the minimum value for the grammar task was found to be 3. We also calculated the standard deviation (SD) for all the task categories of the whole participant ratings. Overall, the SD was found to be 0.99, 0.99, 0.63, and 0.94 for conversation, writing, grammar, and vocabulary, respectively.

The skewness distribution of all the task categories, conversation (−0.61), writing (−1.08), grammar (−0.13) and vocabulary (−1.71), do not show a normal distribution among the participants. However, since the values are more negative and left-skewed, then the majority of the participants are closer to the higher end of the scale (closer to 5) as compared to the lower end of the scale (close to 1). The kurtosis distribution for the writing and grammar task categories shows the normal distribution among ChatGPT’s performance ratings as the values are 0.91 and 0.17, respectively. This shows that the peak of distribution is neither too sharp nor too flat as compared to the normal distribution. Furthermore, the remaining two kurtosis values for listening (−0.15) and reading (3.53) tasks do not show the normal distribution among the participants’ ChatGPT performance ratings. The value −0.15 shows the relatively flat peak/flatter distribution than normal distribution indicating a lighter-tailed distribution. The value 3.53 shows a higher peaked concentration as compared to the normal distribution indicating a gathering of a higher part of data points around the overall mean.

**Figure 7:**
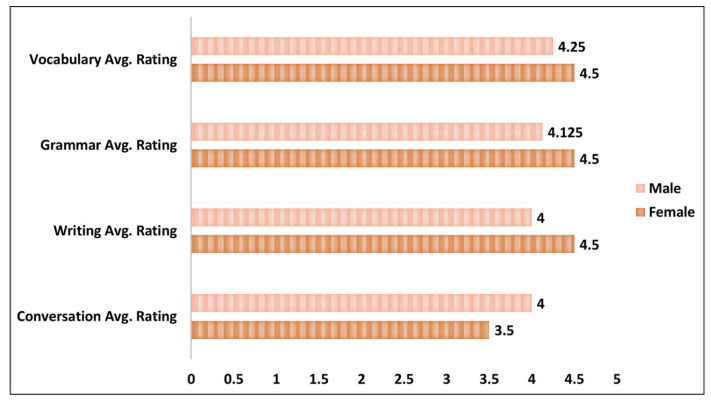
Gender-based task performance rating scores.

Figure 7 shows the distribution of participants’ average ratings related to the performance of ChatGPT for each of the task categories based on the gender attribute. The female group is higher in terms of average performance rating from ChatGPT for three task categories—vocabulary, grammar, and writing—with average scores of 4.5 for all the task categories. For the conversation task, the male group has more average performance rating as compared to the female group.

**Figure 8:**
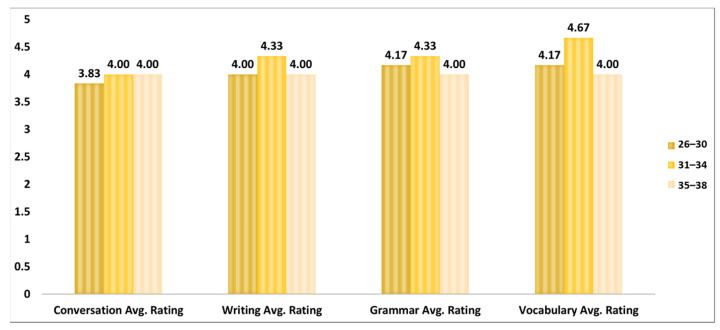
Age-group-based task performance rating scores.

Figure 8 depicts the average rating of ChatGPT’s performance for all the task categories based on three age groups. The age group 31–34 has the highest average scores of performance rating for all the task categories followed by the 26–30, 35–38 age groups, respectively. For the age group 31–34, the highest score for participants is in the vocabulary task (5) followed by the grammar (4.33), writing (4.33), and conversation (4) tasks. The age group 26–30, which contains 60% of the participants, also has the maximum average score for the vocabulary (4.17) task followed by the grammar (4.17), writing (4), and conversation (3.83) tasks. The lowest average scores’ age group is the 35–38, which shows slightly different patterns in the average scores. All the task categories have the same average performance rating of 4.

**Figure 9:**
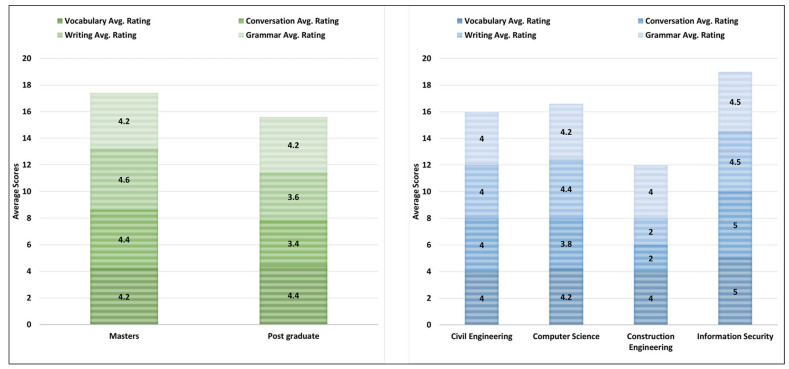
Education level, department-based task performance rating scores.

Figure 9 shows the average rating scores for all tasks performed with ChatGPT based on participants’ current education level and the departments they belong to groups. For the vocabulary task, the participants with postgraduate group have the highest average score of 4.4 out of 5 as compared to master’s group. For the conversation and vocabulary tasks, the participants with master’s have higher average rating scores ranging from 4.4 to 4.6 as compared to the postgraduate group. Finally, for the writing task performed with ChatGPT, both education level groups have the same average rating score of 4 out of 5.

Furthermore, the participants from the Information Security department group have the highest average rating scores for all the tasks performed with ChatGPT followed by the Computer Science, Civil Engineering, and Construction Engineering departments groups. The highest average rating score was found to be from the participants of the department of Information Security for the vocabulary and conversation tasks. However, the lowest average rating score was from the participants of the department of Computer Science for the conversation task performed with ChatGPT.

In order to assess the usability of ChatGPT for the above-mentioned tasks, we asked the participants to fill two questionnaires in the post-experiment questionnaire form. The questionnaires are (1) USE and (2) SUS. The questionnaires and the tasks have been already described in detail above. The participants rated their feedback for each of the questions in both questionnaires from 1 to 5 (i.e., 1 being minimum and 5 being maximum skill level). Since these are the standard questionnaires to assess the usability of any technical system; hence analyzing the participants’ feedback help to understand the usability of ChatGPT for our specific study. To quantify the participants’ feedback scores, we performed the steps below:

1. For the SUS questionnaire, we sum up the feedback score given for each of the questions for all 10 questions. This summation is calculated against each individual participant response and used as the base feedback score.
2. For the USE questionnaire, we sum up the feedback score given for each of the questions for all the questions in each aspect (i.e., ease of use, ease of learning, usefulness, and satisfaction). The summation is calculated against each individual participant response for each of the sections. This score is used as the base feedback score to perform the data analysis.
3. Hence, in the end we assess the usability of ChatGPT for English language learning in terms of total five aspects. (1) SUS—System Usability Scale, (2) Usefulness, (3) Ease of Learning, (4) Ease of Use, and (5) Satisfaction.

The descriptive statistics for all above five aspects are provided in Table 5, where we have shown the SD, mean, min, max, kurtosis, and skewness values for each of the sections in the participants’ feedback for usability of ChatGPT.

The overall mean of sum of participants’ feedback scores for using ChatGPT in terms of five aspects were found to be 29.90 out of 50 for SUS, 30.80 out of 40 for usefulness, 43.30 out of 55 for ease of use, 17.50 out of 20 for ease of learning, and 29.70 out of 35 for satisfaction. Furthermore, the (min, max) scores pairs from the participants’ responses are (26, 35), (31, 54), (13, 20), (19, 38), and (21, 35) for the SUS, ease of use, ease of learning, usefulness, and satisfaction, respectively. The SD was found to be 3.00, 7.60, 5.35, 2.36, and 3.88 for SUS, ease of use, usefulness, ease of learning, and satisfaction, respectively. 

The skewness distribution of participants’ feedback for all the five aspects of usability is also discussed in Table 5. The SUS aspect has overall skewness of 0.15, which shows a positive skewness but almost equal to symmetrical. For the rest of the aspects, the skewness is negative skewed with −0.52 (ease of use) as moderate and −1.12 (usefulness), −1.00 (ease of learning), and −1.07 (satisfaction) as high skewed. The more tendency towards moderate and high negative skewness shows most participants are closer to the higher end of the scale (closer to 5) as compared to the lower end of scale (i.e., 1). The kurtosis distribution for all the five aspects of the usability tends to be more towards the flatter curve as compared to the normal or high peak curves.

**Figure 10:**
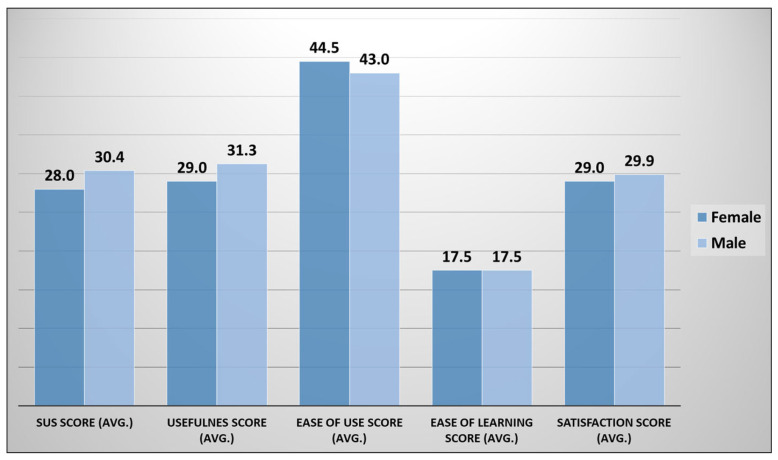
Gender-based SUS, USE questionnaire response distribution.

Figure 10 shows the distribution of participants’ feedback for all the five aspects of usability based on gender attributes. The male group is higher in terms of average score for three aspects e.g., SUS, usefulness, and satisfaction with overall scores of 30.4 out of 50, 31.3 out of 40, and 29.9 out of 35, respectively as compared to the female group. For the ease of use aspect, the female group has a higher average score of 44.5 out of 55 as compared to the male group. For the ease of learning aspect, both gender groups have the same average score of 17.5 out of 20.

**Figure 11:**
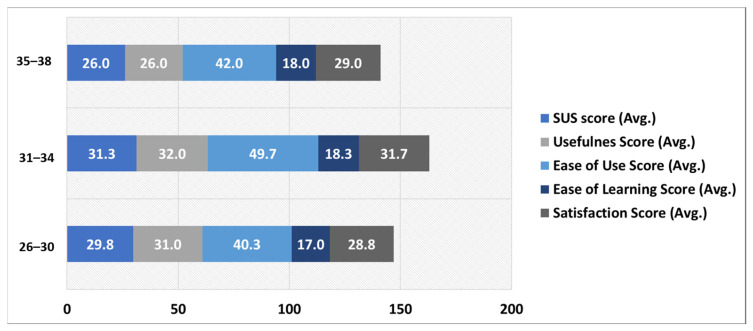
Age-group-based SUS, USE questionnaire response distribution.

Figure 11 depicts the distribution of participants feedback for all the five aspects usability based on age group attribute. The age group 31–34 has the highest average scores for all the five aspects followed by the 26–30 and 35–38 age-groups, respectively. In the age group 31–34, the highest average score of the participants is for the ease of use aspect (49.7 out of 55) followed by usefulness (32 out of 40), satisfaction (31.7 out of 35), SUS score (31.3 out of 50), and ease of learning (18.3 out of 20) aspects. The age-group 26–30, which contains 60% of the participants, also has the highest average score for the ease of use aspect.

**Figure 12:**
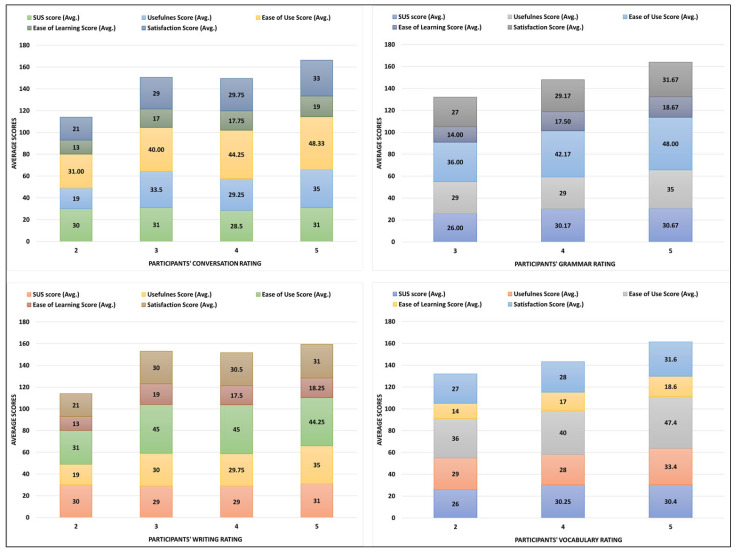
Impact of ChatGPT’s task performance rating on feedback for ChatGPT’s usability.

Figure 12 depicts the implication/relationship of participants’ ratings for all the tasks performed with ChatGPT versus the participants’ rating for all the five aspects of usability. For the conversation task, the highest average score among all the usability aspects is for the ease of use aspect with 48.33 out of 50. This shows that the participants consider ChatGPT easy to use for doing the conversation task among all the other tasks performed with ChatGPT. The same pattern is identified for performing the grammar and vocabulary tasks where the highest average score is rated for the ease of use usability aspect. However, for the remaining task (i.e., writing), the rating pattern is slightly different.

Next, for the ease of learning aspect, the participants with highest rating of 5 for all the tasks performed with ChatGPT have also given the highest rating score for this usability aspect.

**Figure A1:**
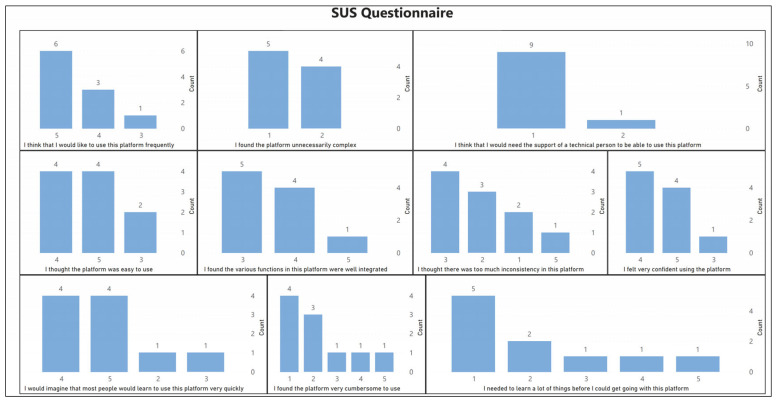
Individual participant feedback for SUS questionnaire.

**Figure A2:**
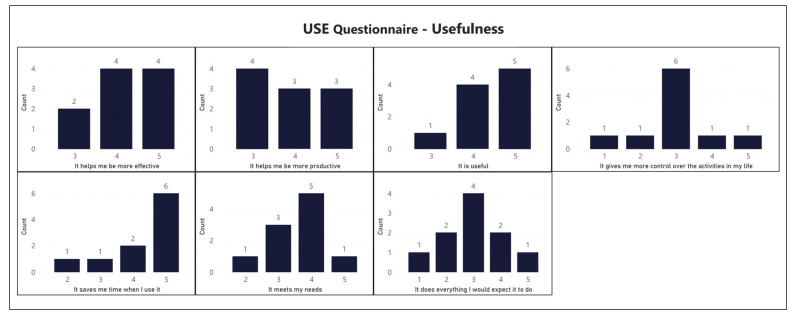
Individual participant feedback for Usefulness–USE questionnaire.

**Figure A3:**
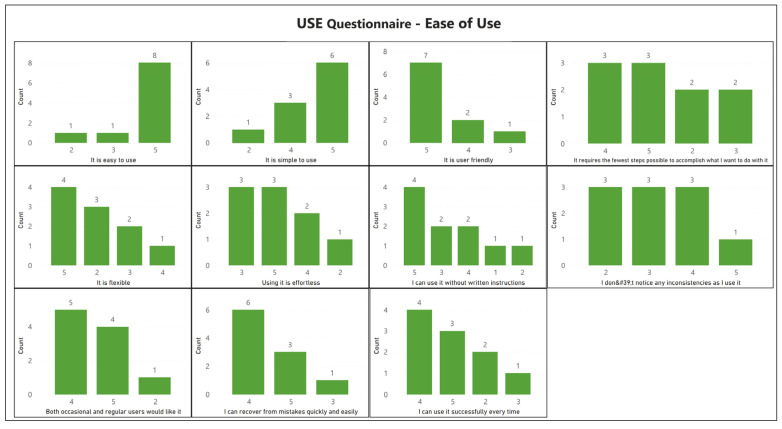
Individual participant feedback for Ease of Use–USE questionnaire.

**Figure A4:**
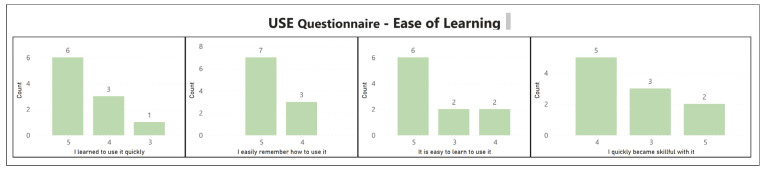
Individual participant feedback for Ease of Learning–USE questionnaire.

**Figure A5:**
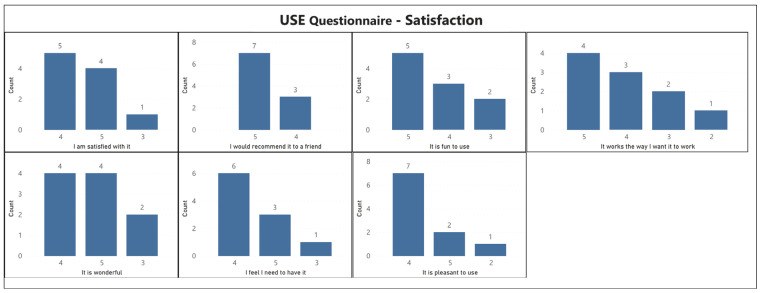
Individual participant feedback for Satisfaction–USE questionnaire.

**Figure A6:**
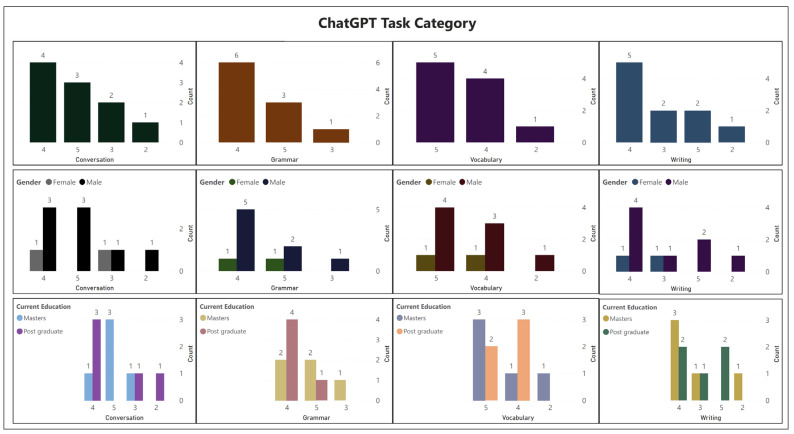
Individual participant feedback for ChatGPT task performance.

Finally, in the Appendix B, Figure A1, Figure A2, Figure A3, Figure A4, Figure A5 and Figure A6 show the individual responses of the participants for each of the questions listed for the SUS and USE questionnaire along with the participants’ rating for each of the tasks performed with ChatGPT for formal English language learning.

## 5. Discussions

In the light of the research objective defined in the introduction section, the results discussed above indicate ChatGPT as a potential resource for formal English language learning. The usefulness of ChatGPT from the participants’ responses in the USE questionnaire indicates that ChatGPT is a useful tool for language learning. The most popular aspect from the USE questionnaire for the participants was ease of use. This means that the participants perceived ChatGPT as user-friendly and easily accessible for the language learners. The effortless, seamless, and friendly interface of the tool contributes to an excellent user experience (direct participant feedback in the form of open text as part of the questionnaire). The satisfaction score from the participants showed a positive experience with ChatGPT. The language learners appreciated the model’s prompt while performing the English language tasks. The participants’ response for the SUS questionnaire showed that the participants perceived ChatGPT as a very usable tool for the English language learning. Most of the participants’ ratings were on the higher end of the Likert scale, which shows participant satisfaction with the system’s usability in terms of ease of learning, ease of use, and usefulness. In summary, the USE and SUS questionnaire participants’ responses provide valuable insights on the usability of ChatGPT for formal English language learning. The positive perceptions for usefulness, ease of use, use of learning, and satisfaction hold a promising and practical implication of ChatGPT for language learners. Furthermore, considering user feedback (related to the interface, or to the quality of responses, etc.) and incorporating consequential measures into further development and improvement of ChatGPT can highly evolve the tool for the better in terms of its overall usability for the English language learners. 

In addition, as the results of this study show, using ChatGPT for English learning has a big potential since learners admitted that ChatGPT can expand their vocabulary, enhance their grammatical and syntactical structures, and thus improve their written and conversational skills [28,29,41]. Students can ask questions, share their thoughts, and discuss various topics, which reflects real-life dialogues and makes them motivated to learn a foreign language (cf. [28,29]). Moreover, ChatGPT can evaluate students’ tasks and can translate texts from one language to another (cf. [42]). Overall, ChatGPT can be used for developing learners’ language skills, scaffolding the learning process by providing feedback to students on their language use and performance. It seems to act as a support tool for practicing a foreign language (cf. [43]).

Based on the in-depth assessment of the usability questionnaire responses, several recommendations emerge for using ChatGPT for English language learning.

1. The language learners and the educators should explore the new methods of integrating this tool to enhance language learning experience.
2. The language learners should try to integrate this tool into instructional practices.
3. The developers of these tools should also consider user-centered design principles to make the language learners accomplish their tasks with as few steps as possible. This is based on the participant responses in the ease-of-use aspect for ChatGPT.
4. The developers can try to continuously improve the user interface, and more interactive elements to increase the satisfaction of the end-users.

There are several limitations of this study related to the fact that the evaluation was conducted only by a very specific group of university students who study computer science; therefore, their view of the topic can be somewhat different from university students who, for example, study English as a second language as their major. It would also seem essential to conduct a similar study in a different cultural context where English as a second language is not at a high level among university students. 

## 6. Conclusions

This paper conducted an exploratory study for assessing the usability of ChatGPT for formal English language learning using a post-questionnaire-based approach. There were, in total, 10 participants recruited to perform different language learning tasks with ChatGPT with diverse demographics attributes such as age, gender, education, English language level, nationality, and field of education. The findings from the participant responses indicated the potential of ChatGPT for automatically generating coherent and correct responses. The conversation interface of ChatGPT allowed interactive dialogues and writing in a natural way. Furthermore, the study also identified several limitations of ChatGPT in the context of language learning and provided specific recommendations. However, it is important to understand that ChatGPT should not replace the human instructions in the language learning, rather that it can be used by language learners with human instructions to fully develop language learning skills. There can be more future directions of this research study where similar a kind of activity can be performed with students/participants from different cultural contexts.

## Figures and Tables

**Table 1:** List of traditional digital language learning technologies (TDLLT).

Category	Description	Example of Software/Application	Purpose
Language learning software	These are standalone computer/mobile software programs.	Rosetta Stone Duolingo Babbel [15]	This software usually covers a comprehensive experience of language learning by using interactive lessons, vocabulary, exercise, pronunciation practice, grammar explanations, etc.
Language learning websites	These are online platforms dedicated to language learning with a wide range of resources, features, etc.	Memrise Busuu FluentU	These websites most of the time offer organized lessons, interactive tasks, quizzes, forums for language learners to interact with each other, etc.
Language exchange websites	These websites create an exchange platform for the language learners. Language learner for the specific target language can practice/learn language together with the native speaker of the target language.	Tandem or HelloTalk [16]	These websites provide different ways for language learners to connect with each other using messaging, video or voice calls, etc.
Flashcard Apps	These applications are used to help learners to memorize vocabulary and phrases through repetition.	Anki Quizlet [17]	These applications make users to create their own flashcards as well as to use pre-made combinations.

**Table 2:** List of emerging language learning technologies (ELLT).

Category	Description	Example of Software/Application	Purpose
AI-based language learning apps	These language learning apps offer personalized learning experience by incorporating artificial intelligence (AI) techniques.	Babbel [18] Duolingo [19] Memrise [20]	The apps analyze users’ performance and progress. Being based on pattern understanding, these apps provide feedback and recommendations and adapt the content and difficulty level for different tasks related to language learning.
VR-based language learning tools	The VR-based tools make learners to involve themselves in a simulated language learning environment by providing a more interactive and realistic learning experience.	Mondly VR VR Speech Flashcard VR for Google Cardboard VR-House [21]	VR-based applications simulate real-life scenarios such as conversation, classroom teaching, group communications, etc. This helps to enable learners to practice language learning skills.
NLP-based language learning platforms	The NLP-based tools are used to help users learn and enhance their language skills by using NLP-powered use cases such as exercise in multiple languages, vocabulary learning, etc.	Google Translate [22] Readlang [23]	NLP-based applications provide interactive exercises and features for effective language learning to enhance language learning activities.
Gamification-based language learning	The gamification-based apps are a type of unique tools which make the language learning experience more engaging and enjoyable. These are developed using game design techniques such as point systems, rewards, badges, and challenges to encourage learners for daily language learning practice.	FluentU MindSnacks [24] Panolingo [25]	The apps allow variety of tasks for language learners through gamification such as translating words, phrases; listening to a phrase and typing what you hear; pronouncing target language words, etc.
Speech recognition-based language learning	The speech recognition-based apps are used to assess learners’ pronunciation accuracy.	Elsa Speak [26] Pronunciation App [27]	These language learning apps analyze speech patterns of the learner and then compare them back to the patterns of native speakers. At the end, these apps provide feedback on pronunciation errors.
Chatbots and conversational AI	The chatbots and conversational AI based language learning tools are used to make learners engage in dialogue/conversation with an AI-powered language assistant.	Chatterbug ChatGPT TalkyLand ELSA Speak HiNative Mitsuku	These tools allow the learners to practice their conversational as well as other skills such as reading and writing.

**Table 3:** Descriptive statistics of participants’ English language level.

	Mean	SD	Min	Max	Skewness	Kurtosis
Speaking	3.50	0.84	2	5	0	0.10
Listening	3.70	0.94	2	5	−0.23	−0.34
Reading	4.30	0.94	2	5	−1.71	3.53
Writing	3.80	0.91	2	5	−0.60	0.39

Note. *N* = 10; Max = Maximum; Min = Minimum; SD = Standard Deviation.

**Table 4:** Descriptive statistics of participants’ rating for the tasks performed with ChatGPT.

	Mean	SD	Min	Max	Skewness	Kurtosis
Conversation	3.90	0.99	2	5	−0.61	−0.15
Writing	4.10	0.99	2	5	−1.08	0.91
Grammar	4.20	0.63	3	5	−0.13	0.17
Vocabulary	4.30	0.94	2	5	−1.71	3.53

Note. *N* = 10; Max = Maximum; Min = Minimum; SD = Standard Deviation.

**Table 5:** Descriptive statistics for USE and SUS questionnaire responses.

	Mean	SD	Min	Max	Skewness	Kurtosis
SUS	29.90	3.00	26	35	0.15	−0.76
Usefulness	30.80	5.35	19	38	−1.12	1.85
Ease of Use	43.30	7.60	31	54	−0.52	−0.91
Ease of Learning	17.50	2.36	13	20	−1.00	0.15
Satisfaction	29.70	3.88	21	35	−1.07	2.16

Note. *N* = 10; SUS = System Usability Scale; Max = Maximum; Min = Minimum; SD = Standard Deviation.

## Data Availability

Due to the ethical, data privacy, and protection concerns, we cannot make the raw data publicly available. However, the individual responses of the participants are already visualized in the Appendix B.

## Appendix A

### Appendix A.1. Task Design Details

**Practice tasks for the students to use ChatGPT for English Language Learning**

Total time: 1 h

Instructions

- For the writing task, please bring two or three English language paragraph texts originally written by you.

**Conversation (15 min)**

Task 1: Start with doing some conversation with ChatGPT to see how much it helps you to learn English language formally in a conversation manner. Below are the possible examples that you can use. You are free to use other ways as well.

- Suggest some topics suitable for the discussion.
- Let’s start the conversation for [topic].
- Let’s have a job interview questions where I will act as a person applying for the job.

Task 2: Also, you can try to ask ChatGPT to generate real-world scenarios for the conversation.

Below are some of the examples but you can use from your own as well.

- Generate a conversation at a police station.
- Re-write the conversation and add a conflict into it.

**Writing (15 min)**

Task 1: Copy your originally written English language paragraph into ChatGPT and perform the below tasks with ChatGPT.

- Rewrite the following paragraph in more formal/informal way.
- Rewrite the following paragraph in a more polite way.
- Rewrite the following text like a formal email.
- Simplify the vocabulary in the following text.

You can perform this task for all the paragraphs which you bring with yourself.

Task 2: Ask ChatGPT to write something for you. Below are some of the examples, but you can use examples from yourself as well.

- Write an email regarding requesting for a fee refund.
- Write an email about asking for rescheduling an email.

**Grammar (15 min)**

Task 1: Copy your originally written English language paragraph into ChatGPT and perform the following tasks with ChatGPT.

- Identify and correct the possible mistakes in the following text.
- Generate a short/simple conversation in a specific [tense].
- Rewrite the following text in passive voice.

**Vocabulary (15 min)**

Task 1: Use different words of your own choice and perform all or some of the following tasks with ChatGPT

- Explain the meaning of [word] in a simple way.
- Generate 20 sentences using the specific [word].
- Explain different contexts of this specific [word].

Task 2: Perform the following tasks with ChatGPT

- Generate a list of most widely used phrasal verbs along with their definitions and examples.
- Generate a list of most commonly used phrases in the business domain.
- Generate a possible list of vocabulary used in the meeting context.

### Appendix A.2. Data Collection Form for Collecting Participants Feedback

1. **Demographics Information**

- Gender (Male, Female, or Other)
- Age
- Current Education Level
- Department
- Previous English language knowledge level

2. **Please rate your previous English language knowledge level (1–5) for below categories**

- Speaking (1–5)
- Listening (1–5)
- Reading (1–5)
- Writing (1–5)

3. **Please rate the following items from (1–5) based on your experience with ChatGPT (USE Questionnaire)**

**Usefulness**

- It helps me be more effective. (1–5)
- It helps me be more productive. (1–5)
- It is useful. (1–5)
- It gives me more control over the activities in my life. (1–5)
- It makes the things I want to accomplish easier to get done. (1–5)
- It saves me time when I use it. (1–5)
- It meets my needs. (1–5)
- It does everything I would expect it to do. (1–5)

**Ease of Use**

- It is easy to use. (1–5)
- It is simple to use. (1–5)
- It is user friendly. (1–5)
- It requires the fewest steps possible to accomplish what I want to do with it. (1–5)
- It is flexible. (1–5)
- Using it is effortless. (1–5)
- I can use it without written instructions. (1–5)
- I do not notice any inconsistencies as I use it. (1–5)
- Both occasional and regular users would like it. (1–5)
- I can recover from mistakes quickly and easily. (1–5)
- I can use it successfully every time. (1–5)

**Ease of Learning**

- I learned to use it quickly. (1–5)
- I easily remember how to use it. (1–5)
- It is easy to learn to use it. (1–5)
- I quickly became skillful with it. (1–5)

**Satisfaction**

- I am satisfied with it. (1–5)
- I would recommend it to a friend. (1–5)
- It is fun to use. (1–5)
- It works the way I want it to work. (1–5)
- It is wonderful. (1–5)
- I feel I need to have it. (1–5)
- It is pleasant to use. (1–5)

4. **Please rate the following items from (1–5) based on your experience with ChatGPT**

- I think that I would like to use this platform frequently. (1–5)
- I found the platform unnecessarily complex. (1–5)
- I thought the platform was easy to use. (1–5)
- I think that I would need the support of a technical person to be able to use this platform. (1–5)
- I found the various functions in this platform were well integrated. (1–5)
- I thought there was too much inconsistency in this platform. (1–5)
- I would imagine that most people would learn to use this platform very quickly. (1–5)
- I found the platform very cumbersome to use. (1–5)
- I felt very confident using the platform. (1–5)
- I needed to learn a lot of things before I could get going with this platform. (1–5)

5. **Please rate your satisfaction (1–5) for different group of tasks you performed with ChatGPT**

- Conversation (1–5)
- Writing (1–5)
- Grammar (1–5)
- Vocabulary (1–5)
